# Comment From the Editor in Chief

**DOI:** 10.1111/obr.70112

**Published:** 2026-02-22

**Authors:** Brian J. Oldfield

**Affiliations:** ^1^ Monash University Melbourne Australia

The Journal is proud to highlight a “Special Issue on the Personalised Management of Obesity,” spearheaded by the BETTER4U Consortium, which has been funded in this regard by the EU.

While there is little doubt that personalized medicine is an aspirational cornerstone of effective prevention, treatment, and ongoing management of obesity, what has been lacking is a cogent and cohesive roadmap toward that endpoint.

It is hoped that the series of papers published in *Obesity Reviews* over coming months under this banner will elucidate the potential of this personalized approach and the course that needs to be followed to achieve it.

I hope that our readership will appreciate the insights derived from this series of papers as much as I have in helping to review them.

Regards


Prof Brian J. Oldfield, PhD,Editor in Chief,*Obesity Reviews*

## Funding

The author has nothing to report.

